# Changes in Cardiovascular Risk Factors After Protocolized Adherence Reinforcement and Treatment Optimization: Results from the OPM Study

**DOI:** 10.3390/jcm15135247

**Published:** 2026-07-05

**Authors:** José Abellán Alemán, Javier Nieto Iglesias, Luis Castilla Guerra, Francisco Fuentes Jiménez, Pablo Sánchez-Rubio Lezcano, Daniel Escribano Pardo, Fernando García Romanos, Rafael Crespo Sabaris, Pablo González Bustos, Fernando Martínez García, on behalf of the Researchers of the OPM Study, José Francisco López-Gil

**Affiliations:** 1 Cátedra de Riesgo Cardiovascular, Universidad Católica de Murcia, 30107 Murcia, Spain; jabellan@ucam.edu; 2 Servicio de Nefrología, Hospital General Universitario, 13005 Ciudad Real, Spain; ljnietoi@gmail.com; 3 Servicio Medicina Interna, Hospital Clínico Universitario Virgen Macarena, Universidad de Sevilla, 41009 Sevilla, Spain; castillafernandez@hotmail.com; 4 Servicio de Medicina Interna, Hospital Clínico Universitario Reina Sofía, 14004 Córdoba, Spain; fjfuentesjimenez@yahoo.es; 5 Servicio de Medicina Interna, Hospital San Jorge, 22004 Huesca, Spain; pablosrl@yahoo.es; 6 Centro de Salud Oliver, 50011 Zaragoza, Spain; danieler49@yahoo.es; 7 Centro de Salud de Santa Catalina, 07013 Palma de Mallorca, Spain; fgarciaromanos@gmail.com; 8 Centro de Salud de Entrena, 26375 La Rioja, Spain; rcrespo@riojasalud.es; 9 Servicio de Medicina Interna, Hospital Universitario Virgen de las Nieves, 18014 Granada, Spain; pabloglezbustos3@gmail.com; 10 Servicio de Medicina Interna, Hospital Clínico Universitario, 46010 Valencia, Spain; fernandoctor@hotmail.com; 11 School of Medicine, Universidad Espíritu Santo, Samborondón 092301, Ecuador; 12 Faculty of Health Sciences, Universidad Autónoma de Chile, Temuco 4780000, Chile

**Keywords:** medication adherence, cardiovascular risk factors, treatment intensification, therapeutic inertia, primary prevention, primary care, implementation science, health services research, Spain

## Abstract

**Background**: Despite evidence-based guidelines for cardiovascular risk management, many patients fail to achieve therapeutic targets. The relative contribution of medication non-adherence versus suboptimal treatment optimization to poor cardiovascular outcomes remains unclear in real-world primary care settings. The aim of this study was to describe changes in cardiovascular risk factor control following protocolized adherence reinforcement combined with physician-driven treatment optimization in high-risk patients. **Methods**: This multicenter, real-world longitudinal study included 789 participants with high or very high cardiovascular risk enrolled from primary care settings across 9 Spanish regions between 2023 and 2025. All participants received a protocolized intervention combining adherence reinforcement and physician-driven treatment optimization. This was a single-arm, pre–post study without a concurrent control group; observed changes therefore cannot be attributed to the intervention alone. Of 789 participants screened, all completed the baseline assessment, and 628 (79.6%) completed the 90-day follow-up. A total of 161 participants (20.4%) were lost to follow-up. Primary outcomes included changes in systolic and diastolic blood pressure, lipid parameters (total cholesterol [TC], low-density lipoprotein cholesterol [LDL-c], high-density lipoprotein cholesterol [HDL-c], triglycerides [TG]), glucose, glycated hemoglobin (HbA1c), and body mass index (BMI) from baseline to 90-day follow-up. Changes were assessed using linear mixed models. **Results**: Among participants with complete paired data (*n* = 453–615 depending on the outcome), significant improvements were observed in most cardiovascular risk factors (HDL-c and HbA1c did not change significantly). Mean changes (95% confidence interval [CI]) were: systolic blood pressure, −9.24 mmHg (−10.41 to −8.06; *p* < 0.001); diastolic blood pressure, −4.75 mmHg (−5.49 to −4.01; *p* < 0.001); LDL-c, −22.29 mg/dL (−25.59 to −19.00; *p* < 0.001); TC, −23.24 mg/dL (−26.73 to −19.74; *p* < 0.001); TG, −16.75 mg/dL (−23.03 to −10.46; *p* < 0.001); fasting plasma glucose, −10.03 mg/dL (−12.61 to −7.46; *p* < 0.001); and BMI, −0.46 kg/m^2^ (−0.58 to −0.35; *p* < 0.001). Linear mixed models including all available data (*n* = 628 at 90-day follow-up) confirmed these findings. No significant interactions were observed between assessment timepoint and sex, age, or overweight/obesity status for most outcomes, except for age-related differences in lipid responses. **Conclusions**: Protocolized adherence reinforcement combined with physician-driven treatment optimization was associated with clinically meaningful improvements in multiple cardiovascular risk factors in high-risk primary care patients. Given the single-arm pre–post design, the observed improvements are associative and cannot establish causality. Residual uncontrolled risk, particularly in lipid management and among older adults, persisted despite active treatment optimization (treatment was modified in 82.0% of participants), consistent with residual suboptimal treatment intensification even after adherence had been reinforced. These findings suggest that achieving optimal cardiovascular risk factor control requires addressing both medication adherence and treatment intensification, particularly in patients with multimorbidity.

## 1. Introduction

Cardiovascular disease (CVD) remains the leading cause of morbidity and mortality globally, presenting a formidable challenge to healthcare systems [1]. In Spain, CVD continues to account for the highest percentage of deaths, underscoring the critical need for rigorous cardiovascular risk (CVR) monitoring in daily clinical practice [2,3]. Despite the availability of robust clinical practice guidelines that define clear targets for blood pressure, lipid, and glycemic control [4,5], a stark contrast persists between recommended goals and real-world patient outcomes. Numerous studies have documented that a substantial proportion of high-risk patients fail to achieve these targets, leaving them vulnerable to recurrent events [6,7].

This gap between guideline recommendations and clinical reality is primarily driven by two distinct but often overlapping factors: therapeutic non-adherence and suboptimal treatment intensification. While non-adherence has been consistently linked to worse cardiovascular outcomes [8], suboptimal treatment optimization, defined as the failure to initiate or intensify therapy when therapeutic goals are not met, represents a systemic failure in healthcare delivery [9,10]. In the context of the Spanish healthcare system, where outpatient and primary care settings often face high patient volumes and time constraints, the risk of suboptimal treatment optimization is exacerbated, particularly in patients with complex multimorbidity and polypharmacy [11].

Recent evidence supports the use of multifaceted interventions, including patient education and digital monitoring, to improve adherence [12,13]. However, distinct from adherence issues, addressing suboptimal treatment optimization requires a fundamental shift in provider behavior. The dynamic nature of CVR profiles demands regular reassessment and timely intensification of therapy [14]. Although protocolized care pathways have shown promise, real-world data quantifying the persistence of suboptimal treatment optimization after adherence barriers have been removed remain limited. Most large-scale registries are observational and do not account for the potential to reverse non-compliance prior to assessing treatment adequacy [15].

This study seeks to bridge this knowledge gap by analyzing a large sample of patients with high and very high CVR in a real-world setting. Unlike purely observational studies, this research evaluates the evolution of control in a prospective cohort where adherence was systematically reinforced, and treatment was optimized when targets were not met. Therefore, the primary objective of the *Objetivo en el Punto de Mira* (OPM) study was to quantify the burden of uncontrolled CVR in a Spanish cohort and, crucially, to determine the extent to which suboptimal treatment optimization persists as the primary barrier to control once therapeutic adherence has been optimized.

## 2. Materials and Methods

### 2.1. Study Design and Setting

This was a multicenter, prospective, quasi-experimental interventional study with a pre-post design, conducted under routine clinical practice conditions in patients with high or very high CVR. Patient recruitment ran from 15 June 2023 to November 2024, and day-90 follow-up was completed by February 2025. Participants were outpatients recruited from 68 primary care centers and specialized hospital outpatient clinics across Spain. Recruitment was performed consecutively by participating physicians under real-world conditions.

The study protocol was reviewed and approved by nine institutional review boards (IRBs) and ethics committees across the participating regions in Spain. The approvals were obtained from: (1) the Research Ethics Committee of *Hospital Universitario San Juan de Alicante* (28 September 2023); (2) the Ethics Committee for Drug Research of Hospital *Clínico Universitario de Valencia* (17 January 2024); (3) the Ethics Committee for Drug Research of the Clinical Research Ethics Committee of Navarra (23 October 2023); (4) the Ethics Committee for Drug Research of Euskadi (Vitoria-Gasteiz, 7 June 2023); (5) the Research Ethics Committee for Drug Research of the Integrated Care Management of *Servicio de Salud de Castilla-La Mancha* (SESCAM), Ciudad Real (18 July 2023); (6) the Medical Research Ethics Committee of *Hospital Clínico Universitario Virgen de la Arrixaca*, Murcia (30 May 2023); (7) the Research Ethics Committee for Drug Research of La Rioja for clinical research with medical devices and research projects (10 October 2023); (8) the Research Ethics Committee for Drug Research of *Complejo Asistencial Universitario de Salamanca* (22 May 2023); and (9) the Research Commission of the Technical Office of Primary Care, Balearic Islands (March 2023). The study was conducted in accordance with the Declaration of Helsinki and all applicable ethical guidelines. All participants provided written informed consent prior to enrollment in the study.

### 2.2. Participants and Eligibility

Recruitment was performed consecutively by participating physicians. Patients were eligible for inclusion if they were adults classified as having high or very high CVR according to the European Society of Cardiology (ESC) guidelines and met at least one of the following criteria:

- Documented established CVD.
- Target organ damage, defined as type 2 diabetes mellitus with duration >10 years or chronic kidney disease (estimated glomerular filtration rate [eGFR] <60 mL/min/1.73 m^2^).
- Severe risk factor elevation: Low-density lipoprotein cholesterol (LDL-c) > 190 mg/dL or grade 3 hypertension.
- A calculated 10-year fatal CVR of ≥5% (high risk) or ≥10% (very high risk) based on the Systematic Coronary Risk Evaluation 2 (SCORE2)/Systematic Coronary Risk Evaluation 2-Older Persons (SCORE2-OP) algorithms.

Exclusion criteria were strictly defined to avoid confounding factors: active collagenopathies, immunosuppressive therapy, severe hepatic insufficiency (transaminases > 2× upper limit of normal), pregnancy, disabling conditions preventing follow-up, or any psychiatric condition compromising informed consent.

### 2.3. Clinical Significance of Observed Changes

To assess the clinical relevance of observed changes, results were interpreted against pre-specified minimum clinically important differences (MCIDs) derived from primary evidence. For blood pressure, a reduction of 5 mmHg in systolic blood pressure (SBP) is associated with an approximately 10% reduction in major cardiovascular events [16]; the clinically meaningful range for diastolic blood pressure (DBP) is 3–5 mmHg. For lipid parameters, each 1 mmol/L (≈38.7 mg/dL) reduction in LDL-c reduces major vascular events by approximately 22% [17]; accordingly, 10 mg/dL represents a conservative MCID for both LDL-c and TC. For glycemic control, a 0.5% reduction in glycated hemoglobin HbA1c corresponds to a clinically meaningful decrement in microvascular complication risk [18]. For body mass index (BMI), a reduction of 1 kg/m^2^ is used as a conservative reference [19]; however, this threshold is less precisely established in terms of direct cardiovascular event reduction, and the observed BMI change (−0.46 kg/m^2^) should be interpreted with corresponding caution.

### 2.4. Sample Size

A formal sample size calculation was performed based on the MCIDs established in the literature for the primary outcomes. A reduction of 5 mm Hg in systolic blood pressure was considered clinically meaningful, given its well-established association with significant reductions in cardiovascular events. For lipid parameters, changes of 10 mg/dL in total cholesterol (TC) and LDL-c were deemed clinically relevant. A change of 0.5% in HbA1c was considered clinically significant, consistent with thresholds used in diabetes outcome studies. For BMI, a change of 1 kg/m^2^ was considered meaningful for cardiovascular risk reduction.

Based on these MCIDs values and assuming standard deviations reported in previous studies, sample size requirements were estimated using paired *t*-tests with 80% power and a two-sided alpha of 0.05. The most conservative estimate corresponded to diastolic blood pressure, requiring 128 participants with complete data at both assessments to detect a 3 mm Hg change. Accounting for an anticipated attrition rate of approximately 20%, a minimum of 160 participants was required.

Of 789 participants screened in the OPM study, all were enrolled and completed the baseline assessment, and 628 completed the 90-day follow-up. A total of 161 participants (20.4%) were lost to follow-up. A total of 453–615 participants (depending on the outcome) had complete data for both assessments and were included in the paired analyses. The final sample size therefore substantially exceeded the minimum required, ensuring adequate statistical power for the primary analyses despite attrition.

### 2.5. Recruitment and Study Procedures

Recruitment was conducted using a consecutive sampling method to minimize selection bias and reflect real-world clinical practice. Each participating investigator included the first 12 eligible patients attending routine consultations who met the inclusion criteria.

The OPM study comprised a 90-day follow-up period with protocolized clinical contacts to reinforce adherence and optimize treatment. All study data were collected by the attending physician using a standardized electronic case report form (eCRF). For cardiovascular risk-factor outcomes, the pre-specified analytic timepoints were baseline (index assessment) and 90-day follow-up, consistent with our companion analysis from the same cohort. The study assessments were as follows:

- Baseline (index assessment): Assessment of eligibility, obtainment of informed consent, and collection of baseline demographic, clinical, and therapeutic data.
- During follow-up (~30 and ~60 days): scheduled clinical contacts for re-evaluation of adherence and treatment adjustment as required. These contacts supported the intervention but were not used as pre-specified analytic timepoints for cardiovascular risk-factor outcomes. a secondary trajectory analysis incorporating these intermediate assessments is described in Section 2.12.
- Final assessment (~90 days; 90-day follow-up): evaluation of cardiovascular risk-factor control and study endpoints.

### 2.6. Data Collection and Measurements

#### 2.6.1. Blood Pressure

Office blood pressure (BP) was measured at each study assessment in accordance with the 2018 ESC/ESH Guidelines recommendations [5]. Measurements were performed using validated semi-automatic oscillometric devices with the patient in a seated position, back supported, and feet flat on the floor, after at least 5 min of rest, following standard technical recommendations [20]. Three consecutive readings were taken 1–2 min apart between 08:00 and 10:00 a.m. to minimize diurnal variability. The mean of the last two measurements was recorded for analysis.

#### 2.6.2. Anthropometry

Anthropometric parameters were assessed using standardized equipment with participants wearing light clothing and no shoes [20].

- Weight and height: Measured using a calibrated scale and stadiometer, respectively.
- BMI: Calculated as weight in kilograms divided by the square of height in meters (kg/m^2^).

#### 2.6.3. Laboratory Parameters

Biochemical parameters, including lipid profile (TC, LDL-c, high-density lipoprotein cholesterol [HDL-c], triglycerides [TG]), fasting plasma glucose [FPG], and HbA1c, were retrieved from the patients’ electronic health records. For baseline assessments, laboratory values obtained within the three months prior to baseline were accepted. When multiple results were available within this window, the most recently obtained value prior to baseline was used. For follow-up assessments, laboratory values obtained up to one month prior to the 90-day follow-up were used. This window was chosen to capture values reflecting the patient’s metabolic status at the time of the follow-up clinical assessment. Samples were processed in each center’s accredited laboratory using standardized enzymatic methods; LDL-c was calculated with the Friedewald equation when TG were <400 mg/dL. Assays were not centralized, which is acknowledged as a limitation; however, within-patient pre–post comparisons are robust to constant between-laboratory offsets.

### 2.7. Therapeutic Adherence and Intervention

Therapeutic adherence was assessed using the Haynes and Sackett test [21].

- Non-adherent patients received standardized adherence reinforcement.
- Adherence was reassessed after 30 days.
- Persistently non-adherent patients were reassessed at 60 days.
- Patients remaining non-adherent were withdrawn from the study.

Patients who were adherent but did not achieve target control values remained in the study and proceeded according to protocol.

For adherent patients outside target ranges, physicians were instructed to optimize treatment at their discretion, including:

- Dose escalation.
- Switching to fixed-dose combinations.
- Monthly follow-up visits were conducted throughout the 3-month period.

The standardized adherence-reinforcement intervention was delivered face-to-face by the treating physician/nurse team and comprised: structured education on the disease and on the rationale for each medication; simplification of the regimen toward fixed-dose combinations where appropriate; identification and resolution of individual adherence barriers; and an agreed action plan. Adherence was re-evaluated with the same Haynes–Sackett instrument at 30 and 60 days; patients who remained non-adherent at 60 days were withdrawn.

### 2.8. Definition of Clinical Control

The criteria were based on international cardiovascular prevention guidelines [4,5,22,23,24], as follows:

#### 2.8.1. Blood Pressure Control

- Age ≥ 65 years: <140/80 mmHg.
- Age < 65 years: <130/80 mmHg.

#### 2.8.2. Dyslipidemia Control

- High CVR: LDL-c < 70 mg/dL
- Very high CVR: LDL-c < 55 mg/dL

#### 2.8.3. Type 2 Diabetes Control

- Age < 80 years: HbA1c < 7%.
- Age ≥ 80 years: HbA1c < 8%.

### 2.9. Outcomes

#### 2.9.1. Primary Continuous Outcomes

Systolic blood pressure and LDL-c were designated as co-primary outcomes; all other parameters were considered secondary/exploratory outcomes.

- SBP (mmHg).
- DBP (mmHg).
- TC (mg/dL).
- LDL-c (mg/dL).
- HDL-c (mg/dL).
- TG (mg/dL).
- FPG (mg/dL).
- HbA1c (%).
- BMI (kg/m^2^).

#### 2.9.2. Derived Variables

- Overweight or obesity: BMI ≥ 25 kg/m^2^.
- Uncontrolled conditions (hypertension, diabetes, dyslipidemia) are defined according to age- and risk-specific thresholds.

### 2.10. Data Management

All data were entered into a predefined Excel database. International data protection standards were followed.

### 2.11. Missing Data

To formally assess the potential for attrition bias, we compared baseline demographic and clinical characteristics between participants who completed the 90-day follow-up (completers, *n* = 628) and those lost to follow-up (*n* = 161; Supplementary Table S2). Non-completers had significantly more favorable baseline lipid profiles (LDL-c 84.3 vs. 101.7 mg/dL, *p* < 0.001; TC 160.0 vs. 177.9 mg/dL, *p* < 0.001; HDL-c 47.6 vs. 51.5 mg/dL, *p* = 0.001) and modestly lower SBP (134.3 vs. 137.9 mmHg, *p* = 0.019), while groups were comparable in age, sex, cardiovascular risk category, glycemia, and BMI. Because completers had higher baseline lipid values, the observed lipid reductions may be slightly overestimated relative to the full cohort; this potential upward bias should be considered when interpreting LDL-c and TC results. A sensitivity analysis using multiple imputation by chained equations (m = 20 datasets; Rubin’s rules) yielded modestly attenuated but directionally consistent and statistically significant estimates for all outcomes (Supplementary Table S1), supporting the robustness of the findings.

### 2.12. Statistical Analysis

Baseline characteristics and follow-up measurements were summarized using descriptive statistics appropriate to the data distribution. Continuous variables were presented as median and interquartile range (IQR) due to potential non-normal distributions, while categorical variables were presented as frequencies and percentages. Missing data were reported for each variable at both assessments. All descriptive analyses were performed separately at baseline and at 90-day follow-up.

To assess within-group changes from baseline to follow-up, we employed two complementary analytical approaches. For participants with complete data at both assessments, paired *t*-tests were used to estimate mean differences (Δ) between 90-day follow-up and baseline, along with 95% confidence intervals (CI). This approach provides an intuitive estimate of the average change but is limited to participants with complete data at both assessments. To account for the longitudinal nature of the data and utilize all available observations (including participants with missing data at one assessment), we fitted linear mixed models (LMMs) with a random intercept for each participant. The general model specification was: Y_ij_ = β_0_ + β_1_ × Assessment_ij_ + b_i_ + ε_ij_, where Y_ij_ represents the outcome for participant i at assessment j, β_0_ is the fixed intercept, β_1_ is the fixed effect of Assessment (coded as “0” for baseline and “1” for 90-day follow-up), bi ~ N(0, σ^2^_β_) is the random intercept for participant i, and ε_ij_ ~ N(0, σ^2^_β_) is the residual error. The estimate of β_1_ represents the mean change from baseline to 90-day follow-up, adjusted for within-participant correlation. Models were fitted using restricted maximum likelihood (REML) estimation. This approach offers several advantages: (1) it accounts for the correlation between repeated measurements within participants, (2) it uses all available data under the missing at random (MAR) assumption, and (3) it provides valid inference even with unbalanced data across assessments.

As a secondary, descriptive analysis, we additionally fitted linear mixed-effects models incorporating all four assessment occasions (baseline, ~30, ~60, and 90-day follow-up), with assessment coded as a four-level categorical fixed effect and a random intercept per participant, to characterize the trajectory of improvement. This secondary model is distinct from the pre-specified primary two-timepoint model (baseline vs. 90-day only) used for the confirmatory main comparisons. Because the two models differ in time-coding structure, the 90-day point estimate may differ marginally between them.

Cardiovascular risk-factor outcomes (blood pressure, lipids, glucose, HbA1c, BMI) were analyzed at two time points only: baseline and 90-day follow-up. Although the protocol included additional clinical contacts during follow-up for adherence reinforcement, intermediate contacts did not provide a complete, balanced set of paired laboratory measurements across participants and were therefore not included in the primary outcome analyses, in line with the pre-specified analytic plan.

To explore whether the intervention effect varied across participant subgroups, we fitted interaction models to test for effect modification by sex, age, and overweight/obesity status. For all interaction models, the main effect of assessment timepoint (β_1_) represents the change for the reference group (males for sex, mean age for age, and no overweight/obesity status), while the interaction term quantifies the differential change for the comparison group.

Model assumptions were evaluated using standard diagnostic procedures. Linearity was assessed through visual inspection of residuals versus fitted value plots. The normality of residuals and random effects was examined using Q-Q plots, and homoscedasticity was evaluated by inspecting residual plots for constant variance across assessments and participant subgroups. Independence of observations was addressed through the inclusion of a random intercept for each participant, which accounts for within-subject correlation, and residuals were further examined to detect any remaining systematic patterns. When model assumptions were substantially violated, appropriate data transformations (e.g., logarithmic transformation for skewed outcomes) or robust standard errors were considered. However, given the primary focus on estimation rather than hypothesis testing and the known robustness of LMMs to moderate departures from normality, transformations were applied only in cases of severe assumption violations.

All analyses were performed using R version 4.4.1 (R Foundation for Statistical Computing, Vienna, Austria). LMMs were fitted using the ‘lme4’ package, with *p*-values calculated using the Satterthwaite method for degrees of freedom via the ‘lmerTest’ package. Statistical significance was set at alpha = 0.05 (two-tailed). However, given the exploratory nature of the interaction analyses and the multiple outcomes examined, results should be interpreted with caution, and emphasis should be placed on effect sizes and confidence intervals rather than *p*-values alone.

Because several effect-modification (interaction) tests were performed, *p*-values for the interaction terms were adjusted for multiple comparisons using the Benjamini–Hochberg false-discovery-rate procedure within each effect-modifier family; adjusted *q*-values are reported alongside nominal *p*-values.

## 3. Results

At baseline, 789 patients were included, of whom 479 (60.7%) were men and 310 (39.3%) were women. According to cardiovascular risk stratification, 318 patients (40.3%) were classified as high CVR and 471 (59.7%) as very high CVR. Overall, 380 patients (48.2%) were in primary prevention and 409 (51.8%) in secondary prevention. The prevalence of cardiovascular risk factors was high: 652 patients (82.6%) had hypertension, 544 (68.9%) had diabetes, and 711 (90.1%) had dyslipidemia. More than half of the cohort (*n* = 426; 54.0%) presented hypertension, diabetes, and dyslipidemia simultaneously (Table 1).

At baseline, 141 participants (17.9%) were classified as non-adherent using the Haynes–Sackett instrument and 646 (82.1%) as adherent (baseline adherence status was missing for 2 participants). Following the standardized face-to-face adherence-reinforcement intervention, 74 of the 141 non-adherent participants reconverted to adherent status by the 30-day reassessment and a further 34 by the 60-day reassessment (108 reconversions in total). Nineteen participants (13.5% of those initially non-adherents; 2.4% of the total enrolled cohort) remained non-adherent at 60 days and were withdrawn from the study per protocol. Accordingly, the analyzed cohort at 90-day follow-up comprised participants who were effectively adherent throughout the observation period.

During the 90-day follow-up, physician-driven treatment optimization was documented across all therapeutic domains (Supplementary Table S3). Treatment was modified in 363 participants (48.5%) in the lipid-lowering domain, in 211 (28.4%) in the antihypertensive domain, and in 204 (28.1%) in the antidiabetic domain. Overall, 647 of 789 participants (82.0%) underwent at least one treatment modification during the study period, confirming that the intervention combined adherence reinforcement with active, protocol-guided treatment intensification. Despite these modifications, a substantial proportion of participants remained above guideline-recommended targets, a pattern consistent with residual suboptimal treatment intensification (therapeutic inertia) as a modifiable barrier to guideline-concordant control. Because the present single-arm design cannot quantify the independent contribution of therapeutic inertia, this remains an inference to be examined in future controlled studies.

As a secondary trajectory analysis, improvements accrued progressively across the intermediate assessments. For SBP, reductions versus baseline were −4.95 mmHg at ~30 days, −6.73 mmHg at ~60 days, and −8.90 mmHg at 90-day follow-up; DBP showed the same progressive pattern (−2.34, −3.66 and −4.62 mmHg, respectively). Lipid and glucose values measured at baseline, ~30 days, and 90-day follow-up followed a comparable downward trajectory (i.e., LDL-c: −12.3 and −20.7 mg/dL; glucose: −6.7 and −10.0 mg/dL at ~30 and 90 days, respectively). Intermediate laboratory data were not collected at the ~60-day contact.

**Figure 1:**
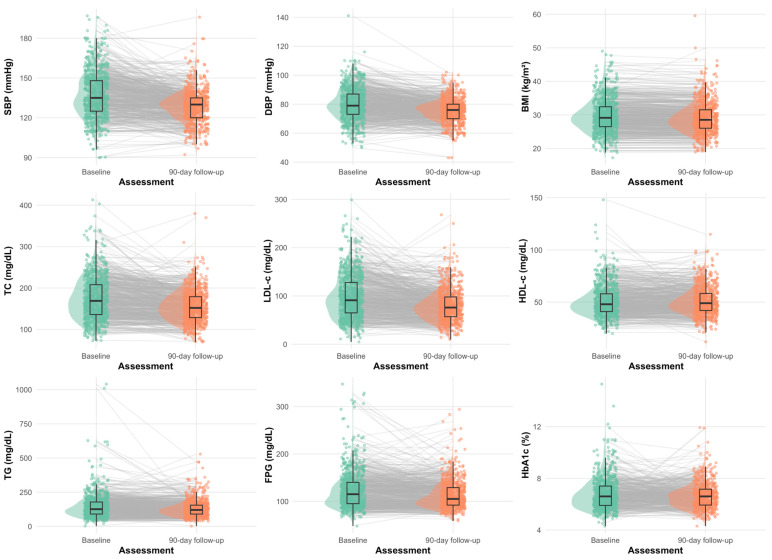
Within-group changes in the outcomes of interest from baseline to 90-day follow-up. Abbreviations: BMI, body mass index; DBP, diastolic blood pressure; FPG, fasting plasma glucose; HbA1c, glycated hemoglobin; HDL-c, high-density lipoprotein cholesterol; LDL-c, low-density lipoprotein cholesterol; SBP, systolic blood pressure; TC, total cholesterol; TG, triglycerides.

From baseline to 90-day follow-up, statistically significant within-group improvements were observed across most continuous cardiovascular risk factors (Table 2; Figure 1). Significant overall reductions were recorded for systolic blood pressure (−8.78 mmHg; 95% CI −9.94 to −7.63), diastolic blood pressure (−4.56 mmHg; 95% CI −5.29 to −3.83), LDL-c (−19.79 mg/dL; 95% CI −23.01 to −16.58), TC (−20.96 mg/dL; 95% CI −24.39 to −17.52), TG (−15.79 mg/dL; 95% CI −21.87 to −9.71), FPG (−9.79 mg/dL; 95% CI −12.32 to −7.26), and BMI (−0.47 kg/m^2^; 95% CI −0.58 to −0.35), all *p* < 0.001. No statistically significant main effects were observed for HDL-c or HbA1c. When evaluated against MCIDs thresholds, the observed changes for blood pressure and lipid profiles were clinically substantial, far exceeding the requirements for clinical significance. Specifically, the reduction in systolic blood pressure represented 175.6% of its MCID threshold (5 mmHg), while LDL-c and TC surpassed their respective thresholds by 197.9% and 209.6% (10 mg/dL), respectively. Diastolic blood pressure also demonstrated robust improvement, achieving 152.0% of its MCID (3 mmHg). Regarding anthropometry, although the reduction in BMI was statistically significant, it reached 47.0% of the MCID threshold (1 kg/m^2^), indicating meaningful progress toward, but not full attainment of, the established threshold for clinical relevance.

**Figure 2:**
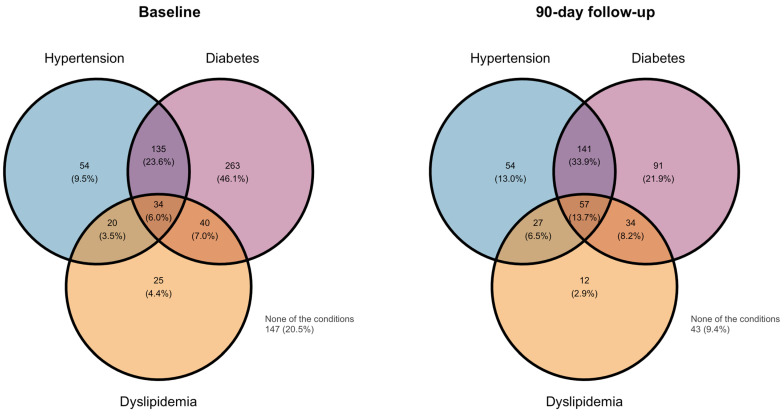
Venn diagram showing the proportion of patients achieving control of hypertension, dyslipidemia, and type 2 diabetes at baseline and at 90-day follow-up.

Improvements in continuous risk factors were accompanied by significant increases in the proportion of patients achieving guideline-recommended control targets. Hypertension control increased from 35.2% at baseline to 59.5% at follow-up (*p* < 0.001). Diabetes control improved from 65.7% to 70.3% (*p* < 0.001), and dyslipidemia control from 19.5% to 27.9% (*p* < 0.001). The proportion of patients with simultaneous control of hypertension, diabetes, and dyslipidemia increased from 5.4% to 12.4% (*p* < 0.001), while the proportion of patients with none of the three conditions under control decreased from 20.1% to 9.4% (*p* < 0.001) (Figure 2).

Interaction analyses are presented in Table 3, Table 4 and Table 5. No significant interactions between assessment timepoint and sex were observed for any outcome (all interaction *p*-values > 0.05), indicating comparable improvements in men and women. After Benjamini–Hochberg correction for multiple interaction tests, no timepoint × sex interaction remained significant; the nominal interaction for diastolic blood pressure (*p* = 0.048) did not survive adjustment (*q* = 0.43). Age significantly modified the effect of the intervention on lipid outcomes. Each 10-year increase in age was associated with attenuated reductions in LDL-c (interaction *B* = 4.37 mg/dL; 95% CI 1.33 to 7.42; *p* = 0.005, *q* = 0.022) and TC (interaction *B* = 5.12 mg/dL; 95% CI 1.87 to 8.37; *p* = 0.002, *q* = 0.019). No significant age interactions were observed for blood pressure, glycemic parameters, TG, or BMI. Overweight/obesity status did not significantly modify changes in most outcomes. However, a significant interaction was observed for BMI, with participants with overweight/obesity showing greater reductions over time compared with those without overweight/obesity (interaction *B* = 0.61 kg/m^2^; 95% CI 0.30 to 0.92; *p* < 0.001; *q* = 0.001). No other timepoint-by–overweight/obesity interactions reached statistical significance.

## 4. Discussion

The results of the OPM study provide a sobering snapshot of the reality of cardiovascular risk management in Spain. Our findings demonstrate that while a protocolized intervention combining adherence reinforcement and treatment optimization led to statistically significant improvements in most continuous risk parameters (SBP, LDL-c, and BMI), the clinical impact, defined as the proportion of patients achieving guideline-recommended targets, remains suboptimal. Only 12.4% of high- and very-high-risk patients achieved simultaneous control of hypertension, diabetes, and dyslipidemia at the end of the 90-day follow-up.

This disconnect between statistical improvement and clinical control suggests that the primary barrier in this cohort may not have been patient non-adherence, but rather insufficient treatment intensification. Since our study design specifically included a standardized adherence reinforcement protocol, and non-adherent patients were either reconverted or excluded, the persistence of uncontrolled risk factors is consistent with residual therapeutic inertia and/or treatment intensification insufficient to reach contemporary targets. This aligns with recent definitions of suboptimal treatment optimization as a system failure rather than solely a provider “delay” [25].

However, it is crucial to distinguish between failure to reach strict guideline targets and the absence of clinical benefit. When evaluated against MCIDs, our results show a highly positive impact. The mean reduction in systolic blood pressure (−8.78 mmHg) nearly doubled the 5 mmHg threshold proposed to reduce major cardiovascular events by approximately 10% [16]. Similarly, the reduction in LDL-c (−19.79 mg/dL) was more than double the 10 mg/dL threshold considered clinically meaningful for vascular protection [17]. This indicates that the intervention successfully reduced the cardiovascular burden, even if it fell short of the aggressive targets (e.g., <55 mg/dL) mandated by modern guidelines.

The most pronounced gap was observed in lipid management. Although LDL-c levels decreased by an average of 19.8 mg/dL and exceeded clinical relevance thresholds, only 27.9% of patients reached the strict targets recommended by ESC guidelines (<55 mg/dL for very high risk) [4]. This finding is consistent with the EUROASPIRE V registry, which highlighted that lipid control is historically the most difficult target to achieve in secondary prevention [6]. Crucially, our interaction analysis revealed that older age was associated with significantly smaller reductions in LDL-c. This suggests a degree of “therapeutic nihilism” or caution in prescribing high-intensity statins or combination therapies to older adults, despite evidence supporting their benefit [11]. As pointed out, overcoming this suboptimal treatment optimization likely requires a shift from stepwise titration to early upstream combination therapy [14].

Interestingly, while diabetes had the highest control rate (70.3%), we observed no significant reduction in mean HbA1c levels (−0.09%, *p* = 0.190). This paradox is likely explained by the baseline characteristics of our cohort. The median baseline HbA1c was 6.6%, suggesting that many patients were already close to target. In this context, suboptimal treatment optimization manifests as “complacency”, or a reluctance to intensify treatment in patients who are “borderline” controlled [9]. Regarding anthropometric parameters, the intervention showed a differential effect based on baseline weight status. Patients with overweight or obesity achieved significantly greater reductions in BMI compared to those with normal weight (interaction *p* < 0.001). This suggests that the adherence reinforcement protocol, which likely included lifestyle counseling, was effective in motivating behavioral changes in the population that needed it most, even if pharmacological intensification lagged behind. Recent evidence emphasizes the importance of personalized cardiovascular risk management, particularly in patients with multimorbidity [26,27]. The variability in treatment response across different risk factors may reflect the complex relationship between polypharmacy, age-related pharmacokinetic changes, and individualized treatment goals.

The age-related differences align with American College of Cardiology (ACC) and the American Heart Association (AHA) guidelines on primary prevention, which emphasize that treatment decisions in older patients should incorporate assessments of functional status and patient preferences [26]. The attenuated response may reflect age-related metabolic changes and polypharmacy considerations. Furthermore, contrary to historical data suggesting that women often receive less intensive CVR management than men [28,29], our study found no significant interaction between sex and the improvement of any risk factor. Both men and women showed comparable reductions in blood pressure and lipids. While this indicates a lack of gender disparity in the effectiveness of the intervention, it also implies that suboptimal treatment optimization affects both sexes equally in this setting.

Our results echo the challenges reported in the global literature but offer a unique insight due to the interventional nature of the study. Unlike purely observational registries, the OPM study actively intervened on adherence. The fact that 87.6% of patients remained uncontrolled in at least one factor despite adherence support reinforces the conclusion that patient compliance alone is insufficient to bridge the gap. The modest reduction in blood pressure (−8.78 mmHg SBP), while clinically meaningful [5], further indicates that physicians often accept “good enough” values rather than striving for optimal control [30].

Furthermore, the principal limitation of this study is its single-arm, pre–post design without a concurrent control group. Consequently, the observed improvements cannot be attributed to the intervention alone. Several alternative explanations cannot be excluded: (i) regression to the mean: patients recruited at a moment of temporarily elevated cardiovascular risk may improve spontaneously regardless of the intervention; (ii) the Hawthorne effect: heightened attention and clinical surveillance inherent to study participation may independently motivate behavior change in both physicians and patients; (iii) increased visit frequency relative to routine care; and (iv) secular trends in cardiovascular risk-factor management. Furthermore, because adherence reinforcement and protocolized treatment optimization were delivered as an inseparable bundle, with the majority of participants receiving at least one treatment modification, the relative contribution of each component cannot be isolated. All findings should therefore be interpreted as associative rather than causal. Randomized controlled trial evidence remains the reference standard; the OPM study provides real-world descriptive evidence to complement and motivate such designs.

## 5. Conclusions

In high-risk primary care patients, a protocolized program combining structured adherence reinforcement and physician-driven treatment optimization was associated with clinically meaningful improvements in blood pressure, lipid parameters, and fasting glucose over 90 days. Given the single-arm pre–post design, causal attribution is not possible; the observed changes may reflect, in part, regression to the mean, the Hawthorne effect, or secular trends. Although treatment was actively optimized in most participants (modified in 82.0% of the cohort), a substantial proportion remained above guideline targets, suggesting that residual suboptimal treatment intensification represents a key, modifiable barrier to guideline-concordant control even after adherence has been optimized. Prospective randomized studies are needed to isolate the individual contributions of adherence reinforcement and treatment optimization, and to determine whether the improvements observed translate into reductions in hard cardiovascular endpoints.

## Figures and Tables

**Table 1:** Descriptive characteristics at baseline.

Variable		
Sex	Male	479 (60.7%)
	Female	310 (39.3%)
Age (years)		66.00 [60.0, 73.0]
	Missing	2 (0.3%)
Height (cm)		167.0 [160.0, 172.0]
	Missing	9 (1.1%)
Weight (kg)	Median [IQR]	81.0 [72.0, 91.0]
	Missing	9 (1.1%)
BMI (kg/m^2^)	Median [IQR]	29.1 [26.5, 32.5]
	Missing	10 (1.3%)
Overweight/obesity	No	132 (16.7%)
	Yes	647 (82.0%)
	Missing	10 (1.3%)
SBP (mmHg)	Median [IQR]	135.0 [125.0, 148.0]
	Missing	1 (0.1%)
DBP (mmHg)	Median [IQR]	79.0 [73.0, 87.0]
	Missing	1 (0.1%)
HDL-c (mg/dL)	Median [IQR]	48.0 [41.0, 58.0]
	Missing	9 (1.1%)
LDL-c (mg/dL)	Median [IQR]	91.1 [65.0, 128.0]
	Missing	9 (1.1%)
TC (mg/dL)	Median [IQR]	169.0 [136.0, 208.0]
	Missing	6 (0.8%)
TG (mg/dL)	Median [IQR]	125.5 [90.0, 178.0]
	Missing	5 (0.6%)
Glucose (mg/dL)	Median [IQR]	115.0 [95.0, 140.0]
	Missing	2 (0.3%)
HbA1c (%)	Median [IQR]	6.6 [5.90, 7.40]
	Missing	67 (8.5%)
Cardiovascular risk	High	318 (40.3%)
	Very high	471 (59.7%)
Uncontrolled hypertension	No	277 (35.2%)
	Yes	509 (64.8%)
	Missing	3 (0.4%)
Uncontrolled diabetes	No	474 (65.7%)
	Yes	248 (34.3%)
	Missing	67 (8.5%)
Uncontrolled dyslipidemia	No	152 (19.5%)
	Yes	628 (80.5%)
	Missing	9 (1.1%)

Abbreviations: BMI, body mass index; DBP, diastolic blood pressure; HbA1c, glycated hemoglobin; HDL-c, high-density lipoprotein cholesterol; LDL-c, low-density lipoprotein cholesterol; SBP, systolic blood pressure; TC, total cholesterol; TG, triglycerides.

**Table 2:** Paired mean differences and linear mixed models estimates.

Variable	*n* (Paired)	Mean (SD) Baseline	Mean (SD) 90-Day Follow-Up	Δ Paired 90-Day Follow-Up−Baseline [95% CI]	*p* (Paired)	Δ LMM 90-Day Follow-Up−Baseline [95% CI]	*p* (LMM)
FPG (mg/dL)	605	123.86 (39.35)	113.83 (31.03)	−10.03 [−12.61, −7.46]	<0.001	−9.79 [−12.32, −7.26]	<0.001
HbA1c (%)	453	6.76 (1.13)	6.67 (1.05)	−0.09 [−0.23, 0.05]	0.190	−0.11 [−0.25, 0.02]	0.084
HDL-c (mg/dL)	601	51.64 (14.71)	51.19 (13.49)	−0.44 [−1.32, 0.43]	0.319	−0.19 [−1.05, 0.66]	0.655
LDL-c (mg/dL)	599	102.62 (44.72)	80.33 (33.54)	−22.29 [−25.59, −19.00]	<0.001	−19.79 [−23.01, −16.58]	<0.001
TC (mg/dL)	599	179.38 (50.01)	156.14 (39.46)	−23.24 [−26.73, −19.74]	<0.001	−20.96 [−24.39, −17.52]	<0.001
TG (mg/dL)	604	146.77 (93.17)	130.03 (62.87)	−16.75 [−23.03, −10.46]	<0.001	−15.79 [−21.87, −9.71]	<0.001
SBP (mmHg)	615	138.09 (16.79)	128.85 (12.32)	−9.24 [−10.41, −8.06]	<0.001	−8.78 [−9.94, −7.63]	<0.001
DBP (mmHg)	615	80.39 (10.76)	75.64 (8.07)	−4.75 [−5.49, −4.01]	<0.001	−4.56 [−5.29, −3.83]	<0.001
BMI (kg/m^2^)	601	29.56 (4.82)	29.10 (4.63)	−0.46 [−0.58, −0.35]	<0.001	−0.47 [−0.58, −0.35]	<0.001

Abbreviations: Δ, mean difference; BMI, body mass index; CI, confidence interval; DBP, diastolic blood pressure; FPG, fasting plasma glucose; HbA1c, glycated hemoglobin; HDL-c, high-density lipoprotein cholesterol; LDL-c, low-density lipoprotein cholesterol; LMM, linear mixed model; *n*, sample size; *p*, statistical significance; SBP, systolic blood pressure; SD, standard deviation; TC, total cholesterol; TG, triglycerides. Notes: Baseline and 90-day follow-up values are presented as mean (SD). Paired Δ represents within-subject mean differences calculated using paired *t*-tests and includes only participants with complete data at both assessments. LMM Δ represents the main effect of assessment timepoint (90-day follow-up minus baseline) estimated from linear mixed models with a random intercept for each participant, using all available observations under the assumption that data were missing at random.

**Table 3:** Interaction: assessment timepoint × sex (female vs. male).

Outcome	Effect	*B* (95% CI)	*p*
FPG (mg/dL)	Δ baseline to 90 days	−9.95 [−13.22, −6.67]	<0.001
FPG (mg/dL)	Interaction: Δ × Female (vs. Male)	0.44 [−4.72, 5.61]	0.867 (*q* = 0.891)
HbA1c (%)	Δ baseline to 90 days	−0.15 [−0.32, 0.02]	0.089
HbA1c (%)	Interaction: Δ × Female (vs. Male)	0.08 [−0.18, 0.35]	0.542 (*q* = 0.743)
HDL-c (mg/dL)	Δ baseline to 90 days	−0.16 [−1.27, 0.94]	0.770
HDL-c (mg/dL)	Interaction: Δ × Female (vs. Male)	−0.12 [−1.86, 1.62]	0.891 (*q* = 0.891)
LDL-c (mg/dL)	Δ baseline to 90 days	−17.97 [−22.11, −13.83]	<0.001
LDL-c (mg/dL)	Interaction: Δ × Female (vs. Male)	−4.62 [−11.17, 1.92]	0.166 (*q* = 0.531)
TC (mg/dL)	Δ baseline to 90 days	−20.16 [−24.61, −15.72]	<0.001
TC (mg/dL)	Interaction: Δ × Female (vs. Male)	−2.05 [−9.03, 4.94]	0.565 (*q* = 0.743)
TG (mg/dL)	Δ baseline to 90 days	−17.56 [−25.42, −9.69]	<0.001
TG (mg/dL)	Interaction: Δ × Female (vs. Male)	4.30 [−8.09, 16.69]	0.496 (*q* = 0.743)
SBP (mmHg)	Δ baseline to 90 days	−9.43 [−10.92, −7.94]	<0.001
SBP (mmHg)	Interaction: Δ × Female (vs. Male)	1.62 [−0.73, 3.98]	0.177 (*q* = 0.531)
DBP (mmHg)	Δ baseline to 90 days	−5.15 [−6.09, −4.21]	<0.001
DBP (mmHg)	Interaction: Δ × Female (vs. Male)	1.50 [0.02, 2.99]	0.048 (*q* = 0.429)
BMI (kg/m^2^)	Δ baseline to 90 days	−0.50 [−0.64, −0.35]	<0.001
BMI (kg/m^2^)	Interaction: Δ × Female (vs. Male)	0.07 [−0.17, 0.30]	0.578 (*q* = 0.743)

Abbreviations: Δ, mean difference; *B*, unstandardized beta coefficient; BMI, body mass index; CI, confidence interval; DBP, diastolic blood pressure; FPG, fasting plasma glucose; HbA1c, glycated hemoglobin; HDL-c, high-density lipoprotein cholesterol; LDL-c, low-density lipoprotein cholesterol; *p*, statistical significance; *q*, Benjamini–Hochberg false-discovery-rate-adjusted *p*-value for the interaction terms; SBP, systolic blood pressure; TC, total cholesterol; TG, triglycerides.

**Table 4:** Interaction: assessment timepoint × age (per 10 years).

Outcome	Effect	*B* (95% CI)	*p*
FPG (mg/dL)	Δ baseline to 90 days	−9.76 [−12.30, −7.23]	<0.001
FPG (mg/dL)	Interaction: Δ × Age (per 10 years)	2.01 [−0.39, 4.42]	0.101 (*q* = 0.227)
HbA1c (%)	Δ baseline to 90 days	−0.12 [−0.25, 0.02]	0.083
HbA1c (%)	Interaction: Δ × Age (per 10 years)	0.01 [−0.12, 0.14]	0.855 (*q* = 0.855)
HDL-c (mg/dL)	Δ baseline to 90 days	−0.18 [−1.03, 0.68]	0.687
HDL-c (mg/dL)	Interaction: Δ × Age (per 10 years)	0.44 [−0.38, 1.25]	0.296 (*q* = 0.444)
LDL-c (mg/dL)	Δ baseline to 90 days	−19.88 [−23.08, −16.68]	<0.001
LDL-c (mg/dL)	Interaction: Δ × Age (per 10 years)	4.37 [1.33, 7.42]	0.005 (*q* = 0.022)
TC (mg/dL)	Δ baseline to 90 days	−20.96 [−24.38, −17.54]	<0.001
TC (mg/dL)	Interaction: Δ × Age (per 10 years)	5.12 [1.87, 8.37]	0.002 (*q* = 0.019)
TG (mg/dL)	Δ baseline to 90 days	−15.82 [−21.90, −9.73]	<0.001
TG (mg/dL)	Interaction: Δ × Age (per 10 years)	2.24 [−3.55, 8.02]	0.448 (*q* = 0.576)
SBP (mmHg)	Δ baseline to 90 days	−8.78 [−9.94, −7.63]	<0.001
SBP (mmHg)	Interaction: Δ × Age (per 10 years)	−0.37 [−1.46, 0.73]	0.514 (*q* = 0.578)
DBP (mmHg)	Δ baseline to 90 days	−4.58 [−5.30, −3.85]	<0.001
DBP (mmHg)	Interaction: Δ × Age (per 10 years)	0.62 [−0.07, 1.31]	0.080 (*q* = 0.227)
BMI (kg/m^2^)	Δ baseline to 90 days	−0.47 [−0.58, −0.36]	<0.001
BMI (kg/m^2^)	Interaction: Δ × Age (per 10 years)	0.08 [−0.03, 0.19]	0.136 (*q* = 0.246)

Abbreviations: Δ, mean difference; *B*, unstandardized beta coefficient; BMI, body mass index; CI, confidence interval; DBP, diastolic blood pressure; FPG, fasting plasma glucose; HbA1c, glycated hemoglobin; HDL-c, high-density lipoprotein cholesterol; LDL-c, low-density lipoprotein cholesterol; *p*, statistical significance; *q*, Benjamini–Hochberg false-discovery-rate-adjusted *p*-value for the interaction terms; SBP, systolic blood pressure; TC, total cholesterol; TG, triglycerides.

**Table 5:** Interaction: assessment timepoint × overweight/obesity (no vs. yes).

Outcome	Effect	*B* (95% CI)	*p*
FPG (mg/dL)	Δ baseline to 90 days	−10.32 [−13.16, −7.49]	<0.001
FPG (mg/dL)	Interaction: Δ × Overweight/obesity (no vs. yes)	4.24 [−2.80, 11.27]	0.238 (*q* = 0.980)
HbA1c (%)	Δ baseline to 90 days	−0.10 [−0.24, 0.04]	0.176
HbA1c (%)	Interaction: Δ × Overweight/obesity (no vs. yes)	0.03 [−0.33, 0.39]	0.863 (*q* = 0.980)
HDL-c (mg/dL)	Δ baseline to 90 days	−0.39 [−1.36, 0.57]	0.426
HDL-c (mg/dL)	Interaction: Δ × Overweight/obesity (no vs. yes)	0.92 [−1.50, 3.33]	0.457 (*q* = 0.980)
LDL-c (mg/dL)	Δ baseline to 90 days	−19.36 [−22.92, −15.80]	<0.001
LDL-c (mg/dL)	Interaction: Δ × Overweight/obesity (no vs. yes)	−3.23 [−12.02, 5.57]	0.472 (*q* = 0.980)
TC (mg/dL)	Δ baseline to 90 days	−21.03 [−24.85, −17.20]	<0.001
TC (mg/dL)	Interaction: Δ × Overweight/obesity (no vs. yes)	−0.36 [−9.86, 9.14]	0.941 (*q* = 0.980)
TG (mg/dL)	Δ baseline to 90 days	−15.82 [−22.61, −9.03]	<0.001
TG (mg/dL)	Interaction: Δ × Overweight/obesity (no vs. yes)	−0.52 [−17.33, 16.30]	0.952 (*q* = 0.980)
SBP (mmHg)	Δ baseline to 90 days	−8.93 [−10.22, −7.64]	<0.001
SBP (mmHg)	Interaction: Δ × Overweight/obesity (no vs. yes)	0.86 [−2.31, 4.03]	0.593 (*q* = 0.980)
DBP (mmHg)	Δ baseline to 90 days	−4.53 [−5.34, −3.71]	<0.001
DBP (mmHg)	Interaction: Δ × Overweight/obesity (no vs. yes)	0.03 [−1.98, 2.03]	0.980 (*q* = 0.980)
BMI (kg/m^2^)	Δ baseline to 90 days	−0.56 [−0.69, −0.44]	<0.001
BMI (kg/m^2^)	Interaction: Δ × Overweight/obesity (no vs. yes)	0.61 [0.30, 0.92]	<0.001 (*q* = 0.001)

Abbreviations: Δ, mean difference; *B*, unstandardized beta coefficient; BMI, body mass index; CI, confidence interval; DBP, diastolic blood pressure; FPG, fasting plasma glucose; HbA1c, glycated hemoglobin; HDL-c, high-density lipoprotein cholesterol; LDL-c, low-density lipoprotein cholesterol; *p*, statistical significance; *q*, Benjamini–Hochberg false-discovery-rate-adjusted *p*-value for the interaction terms; SBP, systolic blood pressure; TC, total cholesterol; TG, triglycerides.

## Data Availability

The datasets generated and/or analyzed during the current study are not publicly available due to institutional restrictions but are available from the corresponding author on reasonable request.

## Supplementary Materials

The following supporting information can be downloaded at: https://www.mdpi.com/article/10.3390/jcm15135247/s1. Table S1. Sensitivity analysis: complete-case vs. multiple-imputation estimates; Table S2. Baseline comparison of completers vs. non-completers; Table S3. Treatment modifications by therapeutic domain during the 90-day follow-up.
