# Supplementary material for: Changes in Cardiovascular Risk Factors After Protocolized Adherence Reinforcement and Treatment Optimization: Results from the OPM Study
Source: J Clin Med. 2026 Jul 5;15(13):5247. doi: 10.3390/jcm15135247 (PMC13362982; doi:10.3390/jcm15135247)
Supplement: Supplementary file 1 [file jcm-15-05247-s001.zip › jcm-4360511-supplementary.pdf]

Supplementary material

Table S1. Sensitivity analysis: complete-case vs. multiple-imputation estimates.

Outcome	n (complete-case)	Complete-case Δ [95% CI]	MI Δ [95% CI]
SBP (mmHg)	615	-9.24 [-10.41, -8.06]	-8.56 [-9.72, -7.40]
DBP (mmHg)	615	-4.75 [-5.49, -4.01]	-4.44 [-5.14, -3.74]
LDL-c (mg/dL)	599	-22.29 [-25.59, -19.00]	-19.63 [-22.64, -16.61]
TC (mg/dL)	599	-23.24 [-26.73, -19.74]	-20.57 [-23.82, -17.32]
TG (mg/dL)	604	-16.75 [-23.03, -10.46]	-15.04 [-20.82, -9.26]
FPG (mg/dL)	605	-10.03 [-12.61, -7.46]	-9.65 [-12.08, -7.22]
HbA1c (%)	453	-0.09 [-0.23, 0.05]	-0.07 [-0.20, 0.06]
BMI (kg/m^2)	601	-0.46 [-0.58, -0.35]	-0.43 [-0.58, -0.29]

Δ, mean change from baseline to 90-day follow-up. Multiple imputation by chained equations (m = 20 imputations) under a missing-at-random assumption; pooled estimates (Rubin's rules). Abbreviations: BMI, body mass index; DBP, diastolic blood pressure; FPG, fasting plasma glucose; HbA1c, glycated hemoglobin; LDL-c, low-density lipoprotein cholesterol; SBP, systolic blood pressure; TC, total cholesterol; TG, triglycerides.

Table S2. Baseline comparison of completers vs. non-completers.

Variable	Completers (n=628)	Non-completers (n=161)	SMD	p
Age (years)	65.7 (10.5)	66.7 (10.7)	-0.096	0.285
SBP (mmHg)	137.9 (16.8)	134.3 (17.4)	0.211	0.019
DBP (mmHg)	80.4 (10.7)	78.4 (11.4)	0.173	0.056
LDL-c (mg/dL)	101.7 (44.5)	84.3 (41.2)	0.405	<0.001
TC (mg/dL)	177.9 (50.2)	160.0 (51.1)	0.355	<0.001
HDL-c (mg/dL)	51.5 (14.7)	47.6 (12.4)	0.293	0.001
TG (mg/dL)	145.8 (92.5)	141.1 (75.7)	0.056	0.507
FPG (mg/dL)	123.4 (39.0)	122.6 (41.5)	0.022	0.811
HbA1c (%)	6.8 (1.2)	6.8 (1.5)	0.024	0.808
BMI (kg/m^2)	29.6 (4.8)	30.4 (5.2)	-0.156	0.089
Sex	Male 379; Female 249	Male 100; Female 61	-	0.751
CVR category	High 249; Very high 379	High 69; Very high 92	-	0.516

Values are mean (SD) for continuous variables and counts for categorical variables. SMD, standardized mean difference (|SMD| > 0.10 indicates a potentially meaningful imbalance). Abbreviations: BMI, body mass index; DBP, diastolic blood pressure; FPG, fasting plasma glucose; HbA1c, glycated hemoglobin; HDL-c, high-density lipoprotein cholesterol; LDL-c, low-density lipoprotein cholesterol; SBP, systolic blood pressure; TC, total cholesterol; TG, triglycerides.

Table S3. Treatment modifications by therapeutic domain during the 90-day follow-up.

**Therapeutic domain**	**Participants modified, n**	**Evaluable, n**	**%**
Lipid-lowering therapy	363	748	48.5
Antihypertensive therapy	211	744	28.4
Antidiabetic therapy	204	725	28.1
At least one modification (any class)	647	789	82.0

Counts reflect participants with at least one documented treatment modification in each therapeutic domain during follow-up, among those evaluable in that domain. The final row reports participants with at least one modification in any therapeutic class. Data derived from the study electronic case report form.

Researchers of the OPM study

José Abellán Alemán (Murcia)
Javier Nieto Iglesias (Ciudad Real)
Pedro J. Tárraga López (Albacete)
Francisco José Fuentes Jiménez (Córdoba)
Fernando García Romanos (Palma de Mallorca)
Jesús Palomo del Arco (Guijuelo)
Alcibíades Segundo Díaz Vera (Pamplona)
María Dolores Martínez Esteban (Málaga)
Alfonso Pobes Martínez de Salinas (Gijón)
Pablo González Bustos (Granada)
Luis Castilla Guerra (Sevilla)
Guillermo Jiménez Portillo (Almería)
Emilio Márquez Contreras (Huelva)
Pilar Segura Torres (Jaén)
Ana Palomo Ruiz (Almería)
Nicolás José Quero Fernández (Almería)
María Cristina Uclés Peña (Almería)
Alicia Tébar Vizcaíno (Almería)
Sergio García González (Almería)
Irene Fernández Carreño. (Almería)
Marina Cejudo Casas (Almería)
María Esther Salguero Cámara (Granada)
Sara Márquez Rivero (Huelva)
María Victoria Quesada Lara (Jaén)
Julio López Fernández (Jaén)
Cinthya Carolina López (Jaén)
Olga Vargas Gómiz (Jaén)
Dulce Nombre Martínez Cámara (Jaén)
Estefanía Ríder Reyes (Jaén)
Pablo Sánchez-Rubio (Huesca)
Cristina Lueza Lampurlanes (Huesca)
María Lalueza Cosculluela (Huesca)
Laura Hernández Camacho (Huesca)
Ana Belén Solano Checa (Huesca)
Ana Isabel Grau Barrull (Huesca)
Maria Victoria Coiduras Sanagustin (Huesca)
Daniel Escribano Pardo (Zaragoza)
Ignacio Durán Sánchez (Zaragoza)
Lucia Cásedas Aguarón (Zaragoza)
Eva María Samatán Ruiz (Zaragoza)
Ana Cristina Martínez Sancho (Zaragoza)
Rafael Crespo Sabaris (La Rioja)
Belén Hernández Ledesma (La Rioja)

Laura Moreno Fernández (La Rioja)
Esther Nieva Porres (La Rioja)
Isabel Sancasimiro Canzano (La Rioja)
Lourdes Canta Castro (La Rioja)
Beatriz López Santa María (La Rioja)
Ángeles Velasco Soria (Murcia)
Esther Uceda Gómez (Murcia)
Ángeles Robles Reyes (Murcia)
José Basilio Gómez Castaño (Murcia)
Francisco Lafuente Salanova (Murcia)
Juan Francisco Martínez García (Murcia)
María José Martí Montoya (Murcia)
Ramón López Guillén (Murcia)
Florentina Rosique (Torre Pacheco)
Daniel Sáenz Martínez (Jumilla)
Jesús Iturralde Iriso (Alava)
Olga López López (Alava)
Amaya Arrieta Aguado (Alava)
Beatriz Barrios Núñez (Alava)
Aránzazu Villa Sanabria (Alava)
Alba Gamboa Serrano (Alava)
Esperanza Moral Berrio (Ciudad Real)
Susana Pedrajas Molina (Ciudad Real)
Patricia Sánchez Escudero (Ciudad Real)
Francisco Javier Alonso Moreno (Toledo)
María Dolores Martínez Malabia (Toledo)
Begoña Rincón Ruiz (Cuenca)
José Antonio Navarro Fernández (Almansa)
Andrea Torres Calatayud (Almansa)
Belén Almudena Tárraga Marcos (Almansa)
Francisco Javier Lucas Pérez-Romero (Casas Ibáñez)
Deyse Helena dos Santos (Casas Ibáñez)
Francisca Molina Escribano (Casas Ibáñez)
Carlos Jesús Cabezas Reina (Toledo)
Laura Cueto Bravo (Toledo)
Borja Alonso Calle (Toledo)
María Ibáñez Cerezo (Toledo)
María Antonia García Rubiales (Toledo)
Marta Romero Molina (Toledo)
Francisco Javier Ahijado Hormigos (Toledo)
Isabel Hervella Durantez (Alicante)
Esperanza González Álvaro ((Alicante)
Greta Asunción Spairani Martínez (Alicante)
Juan Pablo Sánchez Núñez (Alicante)

Fernando Martínez García (Valencia)
Pablo Rodríguez Doyagüez (Guadalajara)
Arianne Aiffil Meneses (Guadalajara)
Jorge Valdés Sotomayor (Cuenca)
Laura Vanessa Blanco Andrews (Ciudad Real)
Emilsi Trinidad Pérez (Alcázar de San Juan)
Freddy Guzmán Ames (Alcázar de San Juan)
Diego Fabián Sidel (Puerto Llano)
José Valderrama Marín (Talavera)
Mª Victoria Guijarro Abad (Talavera)
Enrique Alberto Ruíz Donoso (Toledo)
José Antonio Gómez Puerta (Cádiz)
Aurea Mauren Guevara Bustamante (Chiclana)
Adriana Lizeth Galindo Guarnizo (Chiclana)
Gloria Antón Pérez (Las Palmas)
Xavier Enrique Guerra Torres (Talavera)
